# Intraocular Osseous Metaplasia in Norfolk Black Turkey (*Meleagris gallopavo*): Clinicopathological Characterization of a Rare Avian Condition

**DOI:** 10.1111/vop.70118

**Published:** 2025-11-27

**Authors:** Taina dos Santos Alberti, Andressa Trindade Nogueira, Magale Dallaporta Furquim, Driele Fernanda dos Santos, Rosimeri Zamboni, Eliza Simone Viégas Sallis, Clairton Marcolongo‐Pereira

**Affiliations:** ^1^ Faculdade de Medicina Veterinária Universidade de Cruz Alta Cruz Alta Brazil; ^2^ Faculdade de Medicina Veterinária Universidade Federal de Pelotas UFPel Pelotas Brazil; ^3^ Faculdade de Medicina Veterinária, Centro Universitário do Espírito Santo UNESC Colatina Brazil

**Keywords:** histopathology, intraocular, *Meleagris gallopavo*, ocular pathology, osseous metaplasia, veterinary ophthalmology

## Abstract

**Background:**

Osseous metaplasia is a rare condition characterized by abnormal bone growth in soft tissues. Although documented in mammals, intraocular osseous metaplasia is uncommon in avian species and has been poorly characterized in turkeys ( *Meleagris gallopavo* ).

**Case Presentation:**

An adult Norfolk Black turkey presented with progressive buphthalmos, caseous ocular discharge, and complete vision loss confined to the left eye. Owing to the poor prognosis, enucleation was performed. Gross examination revealed a friable, caseous, yellow‐white intraocular mass with retinal and vitreous involvement. Histopathological examination revealed abundant osteoid matrix and osseous trabeculae, intense neovascularization, heterophilic infiltration, and areas resembling the medullary bone.

**Discussion:**

Histopathological findings support the diagnosis of intraocular osseous metaplasia, which is distinct from normal scleral ossicles in birds. Chronic inflammation and longstanding retinal detachment may trigger differentiation of retinal pigment epithelium (RPE) into osteoblast‐like cells, contributing to osseous metaplasia in the ciliary body. Intraocular bone formation in birds remains poorly understood, and further investigation of its pathogenesis is warranted.

**Conclusion:**

This report describes a well‐characterized case of intraocular osseous metaplasia in a turkey. Recognition of this uncommon condition is essential when considering the differential diagnoses of intraocular masses in avian species, emphasizing the diagnostic value of histopathological evaluation. Further studies are warranted to explore the possible systemic involvement and to elucidate the underlying etiologies, including chronic inflammation and trauma.

## 1 Introduction

Metaplasia is the change from one differentiated (mature) cell type to another differentiated cell type of the same germline [1]. Osseous metaplasia (OM), also known as heterotopic bone formation, refers to abnormal bone growth in muscles and soft tissues [2]. Although the precise etiology of intraocular OM is unknown, it can develop secondary to penetrating trauma, intraocular infections, chronic inflammation, and longstanding retinal detachment [3, 4, 5].

Intraocular OM is often reported in guinea pigs ( *Cavia porcellus* ), where it has a significant prevalence, and in some cases, is associated with high levels of ascorbic acid in the aqueous humor [3]. In humans, intraocular ossification has been correlated with various conditions, including retinal detachment, hemorrhage, fibrovascular proliferation, gliosis, inflammation, trauma, glaucoma, congenital disease, and neoplasia [4, 6]. Osseous metaplasia of the iris is a rare finding in dogs [6]. In cats, it occurs infrequently and is generally associated with severe ocular trauma and post‐traumatic ocular sarcomas. This phenomenon is often related to lens capsule rupture [7]. Intraocular ossification is relatively common in some Galliformes, such as chickens [8], and has also been documented in other avian species [9, 10, 11, 12].

Osseous metaplasia is considered an uncommon finding in turkeys, and reports are particularly limited, likely because ocular pathology is not routinely performed in this species.

This case report describes the clinical, gross, and histopathological characteristics of intraocular osseous metaplasia in a Norfolk Black turkey ( *Meleagris gallopavo* ).

## 2 Case History

**FIGURE 1:**
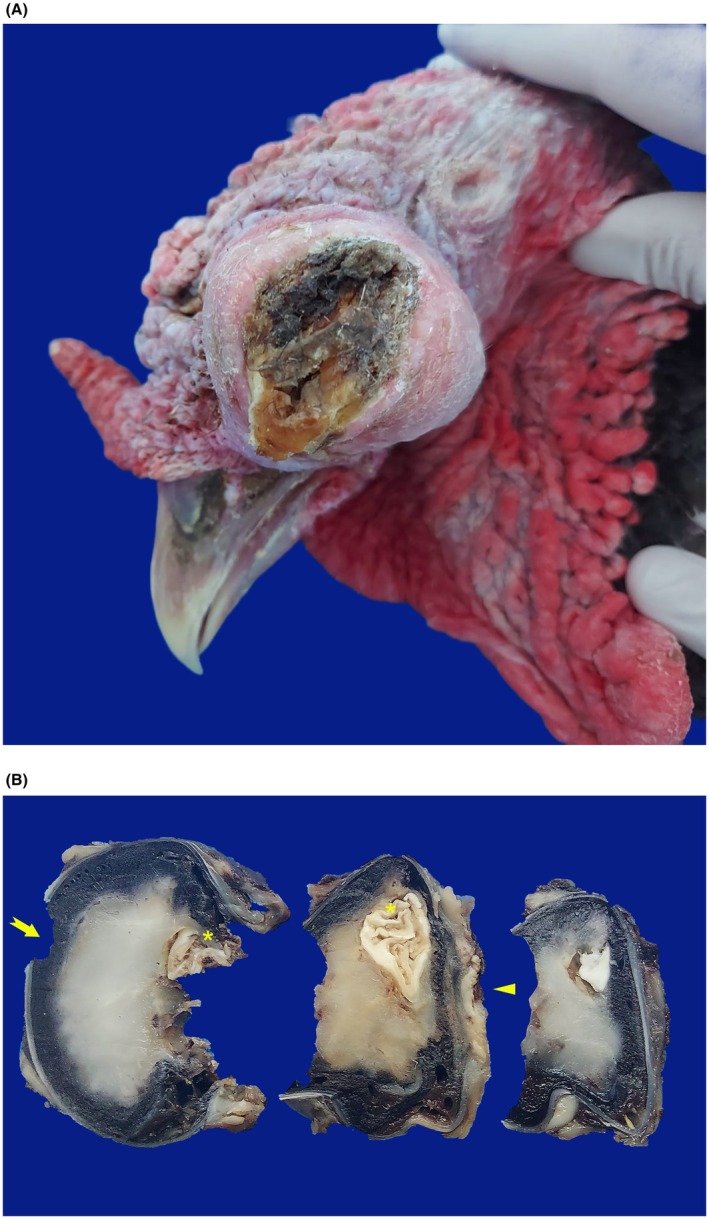
Gross images of intraocular osseous metaplasia in a Norfolk Black turkey ( *Meleagris gallopavo* ). (A) External view of the left eye partially covered by pale tan skin surrounded by a large, dense mass composed of dull, friable, yellow‐brown caseous material. (B) Cross sections of the formalin‐fixed left eye showing an ulcerated and retracted cornea (*arrow*) and a vitreous chamber filled with dense gray‐brown material. A firm, white, dull, irregular mass (*asterisk*) is present near the optic nerve region (arrowhead).

An adult Norfolk Black turkey presented with mild serous discharge in the left eye (OS) that gradually progressed to caseous exudation over several days. Subsequently, the bird developed buphthalmos in the OS (Figure 1A), accompanied by a complete loss of vision within a 1.5‐month period. The OD was clinically and grossly within normal limits. Complete vision loss was diagnosed based on the absence of a menace response and lack of behavioral reactions to visual stimuli. Based on the clinical presentation and poor prognosis of visual recovery, enucleation was performed on the OS of the patient. Unfortunately, the animal died two months after enucleation, and the owner did not authorize necropsy.

The excised globe was fixed in 10% neutral buffered formalin and subjected to gross and histopathological evaluation at the Veterinary Pathology Laboratory. On gross examination, the left eye was partially covered by pale tan skin and surrounded by a dense, yellow‐white, friable mass with a matte, and caseous appearance. The specimen measured 4.5 × 3.8 × 3.5 cm, and the cornea was ulcerated. Upon sectioning, the vitreous chamber was filled with dense, gray‐brown material. Near the optic nerve, an irregular, firm, white mass measuring approximately 1.0 × 0.8 × 0.6 cm was identified extending from the retina to the vitreous body (Figure 1B).

**FIGURE 2:**
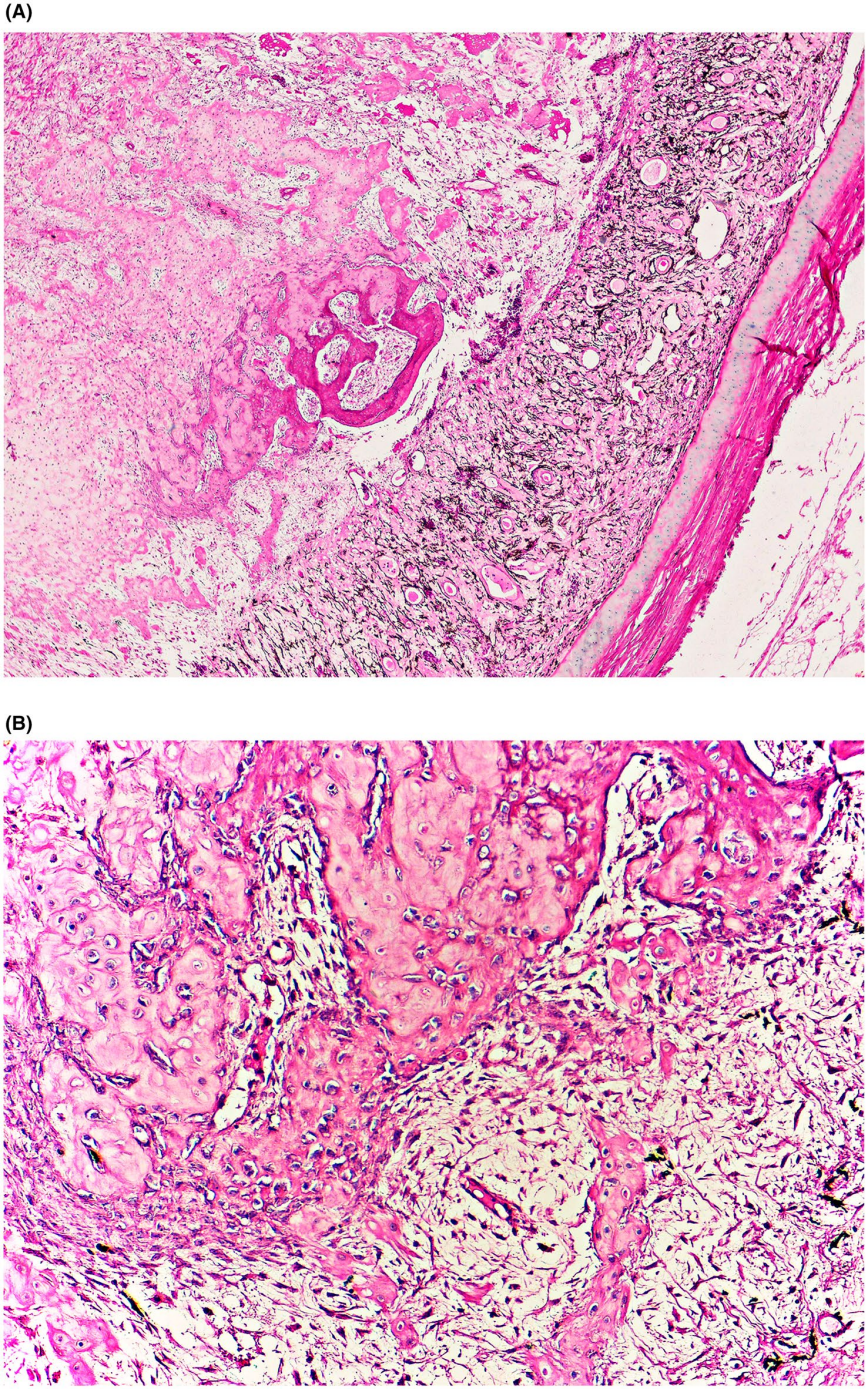
Photomicrograph of intraocular osseous metaplasia in a Norfolk Black turkey ( *Meleagris gallopavo* ). (A) Histological examination of the retina in the left eye revealed a focus of osseous metaplasia, with ectopic bone formation arising within and replacing the retinal tissue. H&E, 10× objective. (B) Abundant eosinophilic bone matrix bordered by plump osteoblastic cells and surrounded by a dense proliferation of fibroblastic mesenchymal cells, consistent with active osseous metaplasia. H&E, 20× objective.

Histopathological examination of the OS revealed a heterogeneous population of polygonal cells associated with osseous trabeculae extending from the retinal pigment epithelium (RPE) into the vitreous body. The trabeculae were well‐organized and, in some areas, contained lacunae filled with inflammatory cells, mimicking the medullary bone (Figure 2A,B). The lesion lacked features of malignancy, such as cellular atypia, abnormal mitotic figures, or infiltrative growth. Marked neovascularization and multifocal hemorrhage were observed within the RPE and ciliary body, accompanied by mild heterophilic infiltration in the vitreous chamber. The cornea exhibited ulceration with associated inflammatory infiltration that extended into the adjacent stroma. The lens was replaced by proliferating polygonal cells and well‐organized osseous metaplasia. The periocular “mass” corresponded microscopically to caseous exudate, composed of amorphous eosinophilic necrotic material admixed with cellular debris and inflammatory remnants, without evidence of infiltration into surrounding soft tissues and skull.

## 3 Discussion

In this case, intraocular osseous metaplasia was confined to the globe with no extension into the adjacent orbital or skeletal tissues. Clinical manifestations included progressive buphthalmos and vision loss. This clinical course is consistent with descriptions of other avian ocular disorders, particularly those involving expansile intraocular tumors [13].

Grossly, the lesion presented as a dense, friable mass with a caseous appearance, occupying most of the globe and obscuring the ocular landmarks. This gross appearance can easily be mistaken for infectious or granulomatous disease, especially in avian patients, where ocular mycobacteriosis and heterophilic granulomas are common differential diagnoses [13]. The presence of necrotic debris, fibrin, and inflammatory infiltrates within the lesion further complicates clinical suspicion, reinforcing the need for histopathological evaluation for a definitive diagnosis.

In this case, histopathology was essential for establishing the diagnosis, revealing heterogeneous cells producing osteoid and a mineralized matrix, accompanied by heterophilic infiltration and stromal neovascularization. These findings are consistent with osseous metaplasia, which involves the formation of mature bone in abnormal locations [2]. The observation of osseous trabeculae with medullary‐like areas is notable and mimics hematopoietic bone marrow, a feature that can be observed in extensive osseous metaplasia [4, 14].

In birds, the presence of scleral ossicles and cartilage as normal components of the eye [7] makes it particularly important to distinguish between normal and metaplastic bone structures. The intraocular location of the lesion, distinct from the scleral ossicles, is crucial for a correct diagnosis. Unlike in mammals, where ocular osseous metaplasia has been more extensively documented [6, 15]. Osseous metaplasia has been rarely reported in birds, except for chickens, where cases are often described [8].

Regarding the pathogenic mechanisms of intraocular osseous metaplasia, Vemuganti [4] suggested that while the inherent high vascularity of the choroid was once believed to predispose it to ossification through the vascular delivery of osteoblasts, it is now proposed that osseous metaplasia primarily results from the osteoblastic transformation of RPE cells. RPE is recognized as a multipotent cell type capable of differentiating into mesenchymal phenotypes, including fibroblasts and osteoblasts [5]. RPE hyperplasia and drusen formation are crucial for intraocular ossification [4, 14] in eyes with chronic retinal detachment. In the present case, histopathological findings revealed that the osseous trabeculae extended from the RPE into the vitreous body, supporting the hypothesis that the metaplastic bone originated from retinal cells. These histological findings not only reinforce the retinal origin of the lesion in our case but also align with previously described mechanisms of osseous metaplasia involving RPE‐derived osteogenic transformation [4].

Drusen, secreted by RPE cells over the Bruch's membrane, may act as a nidus or an inducing agent in the early stages of osteogenesis [4]. Furthermore, preretinal ossification is thought to involve the migration of the RPE from the subretinal space to the retinal surface, often within fibrous membranes or proliferating vitreoretinal masses, indicating multidirectional metaplasia of RPE cells [14]. This process is particularly relevant in cases of longstanding retinal detachment, as observed in humans [15], and is potentially applicable to the present case.

In addition to the RPE, lens epithelial cells may also represent a potential source of cellular transformation in intraocular osseous metaplasia. Epithelial‐to‐mesenchymal transition is a complex biological process that can lead to significant cellular changes, including the acquisition of mesenchymal traits and increased plasticity. This phenomenon has been observed in lens epithelial cells in conditions of ocular trauma or chronic inflammation [16]. In the present case, considering the severe intraocular disruption and corneal ulceration, it is plausible that both the RPE and lens epithelial cells could have participated in the metaplastic response.

In guinea pigs, where intraocular osseous metaplasia is relatively common, high levels of ascorbic acid (vitamin C) in the aqueous humor have been proposed to promote localized ossification [3]. Although comparable data in birds are lacking, this hypothesis raises interesting questions regarding possible species‐specific biochemical factors that may influence OM formation across different taxa.

Trauma is a well‐recognized inciting factor for intraocular osseous metaplasia, particularly in species predisposed to ocular injury, such as birds. Blunt trauma may initiate a cascade of chronic inflammation, retinal detachment, and structural damage to the RPE, thereby creating a permissive environment for osteogenic transdifferentiation. The identification of metaplastic bone in noncontiguous intraocular regions further supports the hypothesis that multiple ocular cell types, including RPE and lens epithelial cells, contribute to heterotopic ossification following injury [8].

This report describes a well‐characterized case of intraocular osseous metaplasia in a turkey. This case highlights the importance of considering non‐neoplastic conditions such as osseous metaplasia in the differential diagnosis of intraocular masses in birds, particularly when clinical signs progress despite medical treatment. It also reinforces the diagnostic value of histopathology for differentiating metaplasia from neoplastic or inflammatory intraocular diseases.

## Author Contributions

T.S.A., A.T.N., M.D.F., D.F.S., R.Z., E.S.V.S., and C.M.‐P. contributed equally to the conception, clinical assessment, pathological analysis, literature review, manuscript writing, and final approval of the submitted version.

## Funding

This work was supported by Coordenação de Aperfeiçoamento de Pessoal de Nível Superior, 001. Fundação de Amparo à Pesquisa e Inovação do Espírito Santo.

## Disclosure

The authors have not used AI to generate any part of the manuscript.

## Ethics Statement

Ethical approval was not required for this case report. The case involved clinical management and surgical removal of the affected eye due to medical necessity. Owner consent was obtained for the use of clinical data and images for publication purposes.

## Conflicts of Interest

The authors declare no conflicts of interest.

## Data Availability

The data that support the findings of this study are available from the corresponding author upon reasonable request.
